# Supplementary material for: Ultrafast vibrational control of organohalide perovskite optoelectronic devices using vibrationally promoted electronic resonance
Source: Nat Mater. 2023 Nov 20;23(1):88–94. doi: 10.1038/s41563-023-01723-w (PMC10769873; doi:10.1038/s41563-023-01723-w)
Supplement: Supplementary file 1 — Supplementary Notes 1–8 and Figs. 1–13. [file 41563_2023_1723_MOESM1_ESM.pdf]

nature materials

Article https://doi.org/10.1038/s41563-023-01723-w

Ultrafast vibrational control of organohalide perovskite optoelectronic devices using vibrationally promoted electronic resonance

In the format provided by the authors and unedited

Contents

Supplementary Note 1: Characterisation of $FAPbBr_3$/$CsPbBr_3$ Devices/Films..2
Supplementary Note 2: Application of the Time Domain phasing/filtering algorithm 4
Supplementary Note 3: Details of the PL-VIPER experiments..6
Supplementary Note 4: Lifetime Analysis of the ν(N-C=N) mode. 7
Supplementary Note 5: Vibrational SFG Response of $FAPbBr_3$ 8
Supplementary Note 6: Wavelength Dependent Control Measurements 10
Supplementary Note 7: Ab Initio Simulations..14
Supplementary Note 8: Experimental origins of the coherent stimulation of the ν(NCN) mode 17

Supplementary Note 1: Characterisation of $FAPbBr_3$/$CsPbBr_3$ Devices/Films

IR Spectra of $FAPbBr_3$/$CsPbBr_3$ Devices/Films

IR spectra of both the $FAPbBr_3$ and $CsPbBr_3$ devices were obtained using an FTIR spectrometer in ATR mode. All spectra were obtained in air and at room temperature. In the case of the $FAPbBr_3$ film, the sample was contacted directly with the ATR crystal, whilst for the $FAPbBr_3$ and $CsPbBr_3$ devices, the semitransparent Ag electrodes were contacted to the ATR crystal.

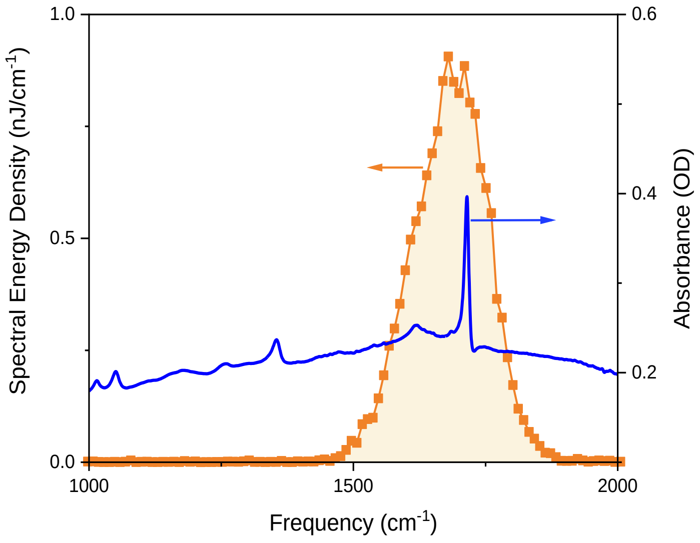

Supplementary Figure 1: IR spectra of an FAPbBr3 device (blue curve), overlaid on the spectrum of the IR pre-excitation pulse employed in this experiment (orange points)

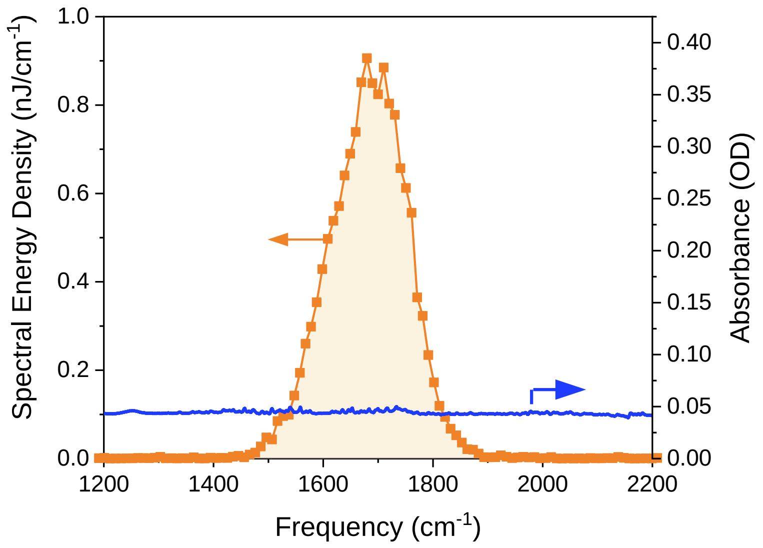

Supplementary Figure 2: IR spectra of a CsPbBr3 device (blue curve), overlaid on the spectrum of the IR pre-excitation pulse employed in this experiment (orange points)

Raman Survey Spectrum of $FAPbBr_3$

The Raman spectra of $FAPbBr_3$ perovskites were measured using a commercial Raman microscope (inVia, Renishaw) with a 50x objective lens (Leica DM 2500 M, NA=0.75). A 633 nm He-Ne (RL633, 17mW) laser was used for measuring both CsPbBr3 and FaPbBr3 perovskites. In order to avoid sample degradation during the measurements, FaPbBr3 film was translated under the laser beam during the measurements with an MS20 encoded stage (Renishaw Inc.) The Raman spectra given in the main text were smoothed using a 10-point Savitzky-Golay filter with a polynomial order of 2.

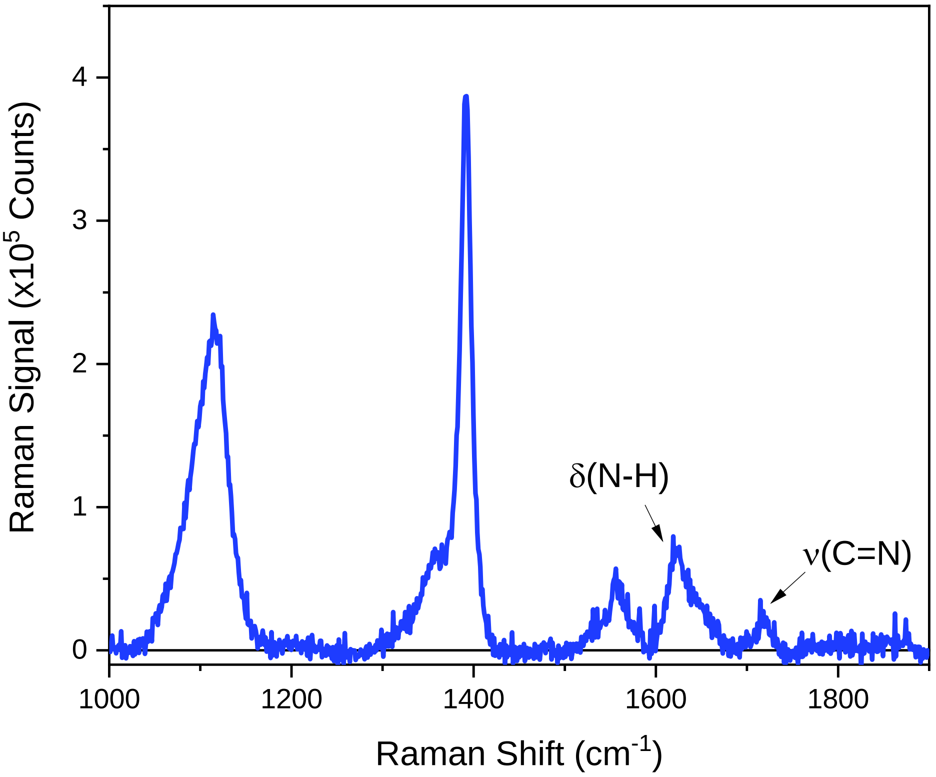

Supplementary Figure 3: Raman spectrum of the FAPbBr3 device employed in our VIPER experiments, with the two peaks relevant to our study labelled.

Supplementary Note 2: Application of the Time Domain phasing/filtering algorithm

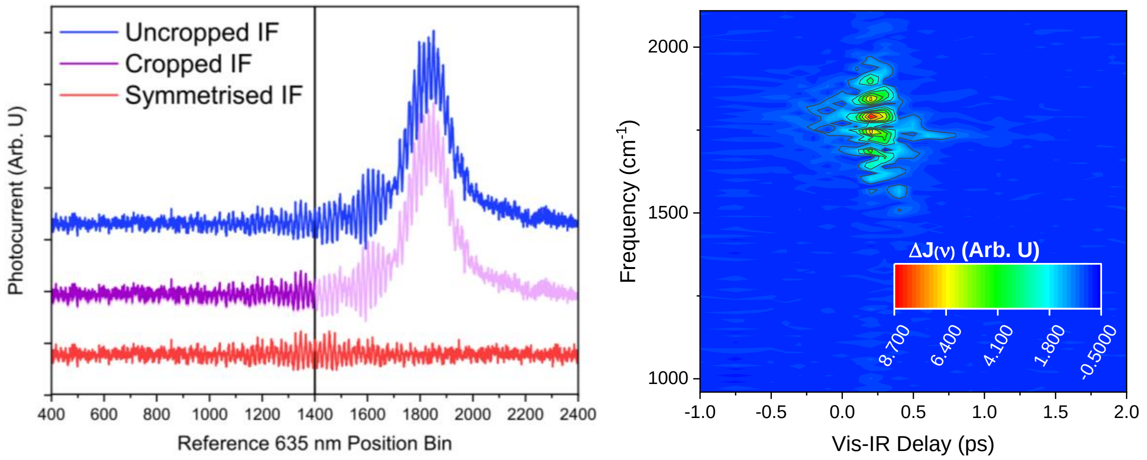

Supplementary Figure 4: (left) Reflection and symmetrisation of the time-domain VIPER interferogram (IF); the position (x) axis is given in multiples of the 635 nm reference laser, (right) 2D map of the PC-VIPER data given in Fig. 3(a) in the main text prior to the symmetrisation process.

To convert the raw time domain interferogram into a frequency domain spectrum, we apply several post-processing methods. Key among these techniques is time domain filtering, which was implemented to reduce the asymmetry present in the time domain data as well as, coherent non-resonant sum-frequency effects. Non-resonant sum-frequency generation during the simultaneous arrival of the visible and IR pulses were found to be highly significant, especially when the desired VIPER signal is weak relative to these phenomena. To reduce the effect of these nonresonant artefacts, the time domain data were cropped to the timeframe before this non-resonant signal becomes significant. To avoid artefacts that would be introduced by cropping the interferogram, the time domain data were then symmetrized by reflection about the point at which the data was cropped, prior to the visible-IR time-zero. To improve the accuracy of this symmetrisation, an additional phasing procedure (outlined in more detail below) was performed.

The time domain processing is outlined visually in Fig. S6: in the case of the unprocessed time domain spectrum, a clear asymmetry is present. This results from the pulse replica from the interferometer's dynamic arm first overlapping with the pulse replica from the static arm (providing the necessary frequency resolution), and then overshooting the static IR pulse and overlapping temporally with the pump pulse. Consequently, the raw time domain data contains both the necessary interference data as well as additional (and unwanted) contributions from interactions between the dynamic IR and visible pulses. To remove these unwanted contributions, we reflect the time domain data at the point where the static and dynamic IR pulse replicas are temporally overlapped, meaning that the undesirable interactions between the dynamic IR pulse and the visible pulse are removed. In order to select the reflection point within the time domain data, we consider the raw output of the Fourier Transform. This transform is complex valued and thus can be expressed in the form

A(ν) + iB(ν), where A(ν) and B(ν) refer to the real and imaginary parts of the overall spectrum. A(ν) and B(ν) represent contributions to the overall spectrum arising from cosinusoidal and sinusoidal components, respectively. Thus, when the reflection point results in a perfect symmetrisation of the data (*i.e.:* at the point of perfect overlap between the static and dynamic IR pulse replicas), the imaginary component B(ν) disappears and the resulting component is solely contained within the real part A(ν). Hence, during post processing, the point which maximizes the real portion of the overall signal was chosen. Following the reflection procedure, the symmetrized interferogram was zero padded to retain the desired 10 cm^{-1} resolution and Fourier transformed.

Supplementary Note 3: Details of the PL-VIPER experiments

The experimental setup for PL-VIPER is largely identical to that of PC-VIPER (outlined above), with several key exceptions, outlined here. Instead of directly connecting a perovskite photovoltaic device into the lock-in amplifier, a home-built objective lens was used to collect photoemission from the perovskite film. The collected photoluminescence was collimated by the objective and subsequently passed to an avalanche photodiode (APD430A, Thorlabs Inc.) *via* an optical fiber. The output of the photodiode was then connected to the lock in amplifier and run with an identical time constant to that of the PC-VIPER experiments.

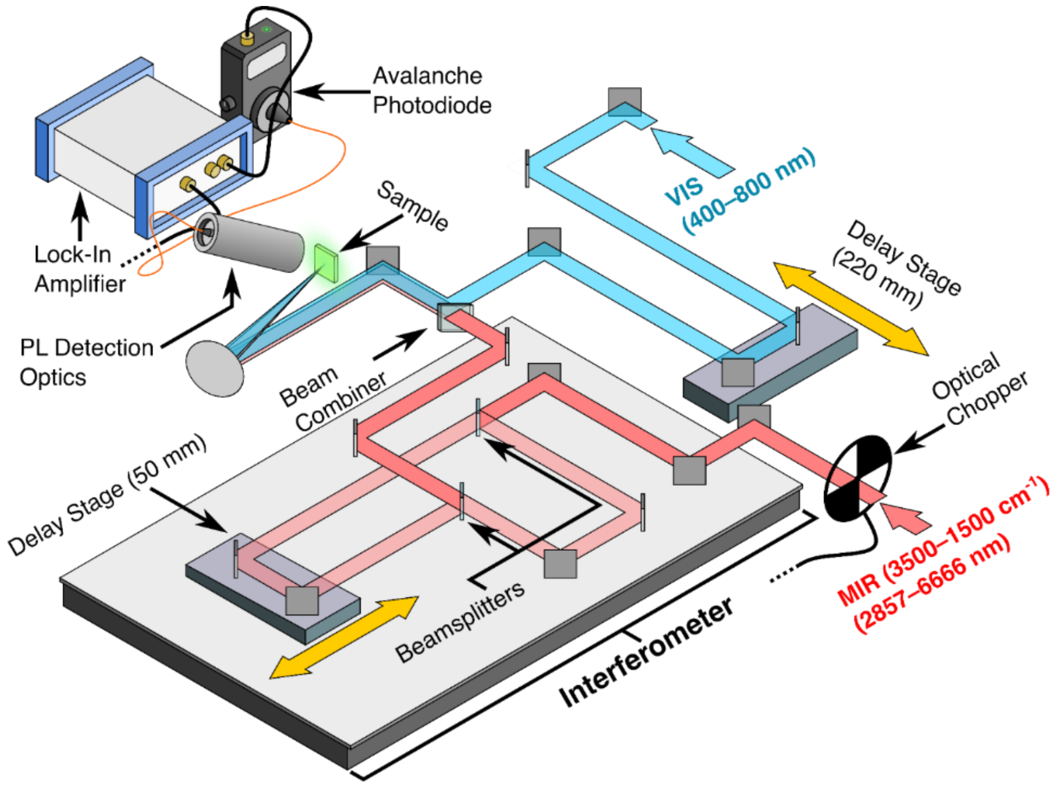

Supplementary Figure 5: Simplified scheme for the PL-VIPER experiments. In contrast to the PC-VIPER setup described in the main text, here we employ $FAPbBr_3$ thin films. The photoluminescence output of the thin film is collected via a home-built objective lens, which is then passed to an avalanche photodiode via an optical fiber. The current output of the photodiode is read using a lock-in amplifier.

Supplementary Note 4: Lifetime Analysis of the ν(N-C=N) mode.

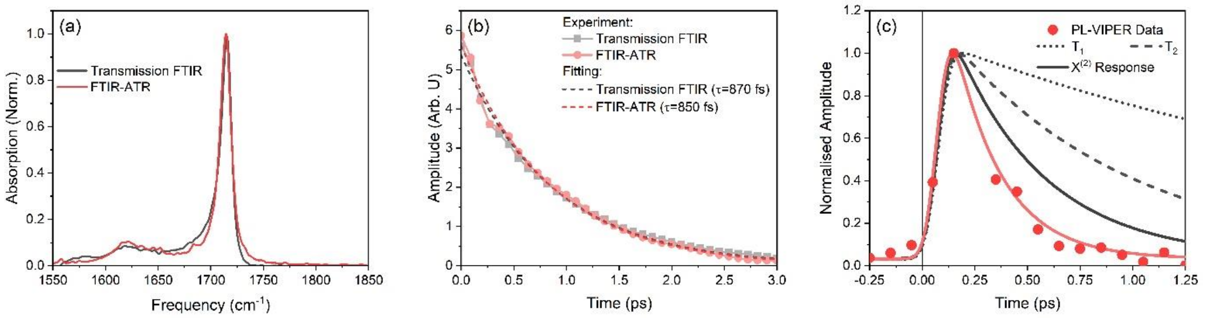

Supplementary Figure 6: Lineshape analysis of the vibrational line of $FAPbBr_3$ at ca. 1720 cm^{-1}. (a) Lineshapes of the 1720 cm^{-1} feature acquired in transmission and ATR mode; (b) calculated polarization relaxation dynamics obtained through Fourier transform of the features in (a). (c) Kinetics of the PL-VIPER signal at 1720 cm-1. The population relaxation (T_1), the free induction decay (T_2, estimated from linewidth analysis) and the estimated $\chi^{(2)}$ signal decay characteristic of the VSFG response are shown for comparison.

We have performed the analysis of vibrational lineshape to estimate the typical timescale of polarisation decay after interaction of the sample with IR pulse. This timescale determines the time evolution of vibrational sum-frequency generation signals (VSFG); specifically, as VSFG is a $\chi^{(2)}$ nonlinear optical process, we expect the time evolution of the VSFG signal to be two times faster than the vibrational free-induction decay. Thus, comparing the estimated polarisation decay dynamics to the experimental data may therefore help to identify the origin of the signals.

We can obtain the polarisation relaxation of the vibrational mode *via* the Fourier Transform of IR absorption lines. Fig. S3(a) displays the ν(N-C=N) mode at *ca.* 1720 cm^{-1} acquired in both transmission and attenuated total reflection (ATR) mode—in both cases we observed relatively minor changes in the vibrational lineshape, although the overall widths of the two lines are similar. Fourier transformation of these vibrational lines yields the relaxation dynamics given in Fig. S3(b), in both cases, the relaxation of the vibrational line is approximately 850—870 fs depending on the measurement method (transmission *vs* ATR).

In both cases, the expected lifetime of the VSFG signal would be half of the free-induction decay time and on the order of 430fs. This is substantially longer than the 280 fs relaxation dynamics observed for our VIPER experiments as shown in Supplementary Figure 6c. To accurately model the expected dynamics of vibrational relaxation given our finite measurement time, we additionally convolute this response with a ~80 fs gaussian instrument response function, leading to the relaxation curves displayed in Fig. S3c.

Supplementary Note 5: Vibrational SFG Response of $FAPbBr_3$

General Experimental Details

VSFG spectra of $FAPbBr_3$ devices and films were acquired using the apparatus in RIKEN. A detailed description of the VSFG spectroscopy systems employed in our experiments can be found elsewhere.[1,2] Briefly, we employed a Ti:Sapphire regenerative amplifier system (Spectra Physics, Spitfire Ace®, 5 W, 80 fs, 800 nm center wavelength) as our primary light source. The mid-IR probe pulse was obtained using a commercial OPA system equipped with a DFG frequency mixer (Light Conversion, TOPAS-prime), and its spectral profile is shown in Fig. S11(c). The mid-IR probe had a pulse-energy of approximately 5 μJ. The mid-IR beam path was purged with dry N_2 (RH < 4%) to reduce the effect of water vapor absorption. The spectrally-narrow visible probe beam was obtained by using the bandpass filter, resulting in ~795 nm (12 579 cm^{-1}) pulses with a bandwidth of ~1.5 nm (~24 cm^{-1}). The pulse energy of the visible probe was approximately 10 μJ. The incident angles of the visible and mid-IR beams on the sample were ~37° and ~42°, respectively. The SF signal was then passed to a polychromator (Princeton Instrument, SP-2300i) equipped with a nitrogen-cooled CCD detector (Princeton Instrument, Spec-10:2KB-dll). The probe beams and the detected SF signal were all p-polarized (*i.e.:* PPP polarization conditions). The typical acquisition time of a VSFG measurement was approximately 3 minutes, containing a total of 3 spectra taken at different lateral positions (differing by 1 mm) to remove any potential spatial inhomogeneity.

VSFG in $FAPbBr_3$

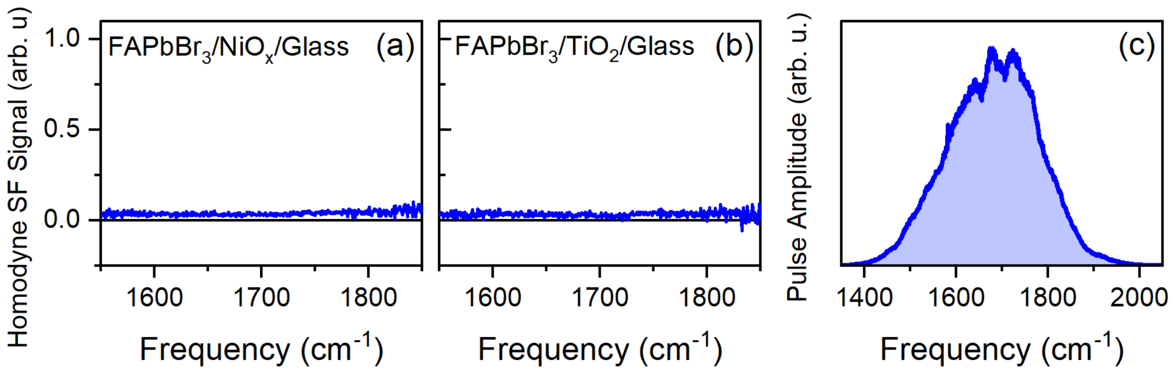

Supplementary Figure 7: VSFG Spectra of FAPbBr3 on (a) NiO_x, and (b) TiO_2; (c) Spectrum of IR pulse employed for VSFG experiments, estimated from nonresonant VSFG signals at an air/z-cut quartz interface.

Homodyne VSFG spectra of $FAPbBr_3$ films are given in Figs. S11(a) and (b). In the case of $FAPbBr_3$ films, we employ both TiO_2 and NiO_x as substrates, as both materials enjoy broad use in solar cells. In both cases, we find no resonant enhancement of VSFG within our measurement window, which covers both the 1600 and 1720 cm^{-1} modes of interest in this study. These findings discount the presence of VSFG within the bulk film and at both the air/$FAPbBr_3$ and the (NiO_x/TiO_2)/$FAPbBr_3$ interfaces, which in turn strongly discounts resonant VSFG emission as the proximate cause of our observed PL-VIPER signal. We note that the experimental system used to obtain these measurements has successfully observed evidence

of VSFG resonances in other perovskite systems, meaning that we expect our instrument to be sensitive to similar effects in our chosen $FAPbBr_3$ systems.[1] Additionally, given the quantitative similarity of both the PL-VIPER signals and PC-VIPER signals in both the time and frequency domains, these results also suggest that VSFG is also not the cause of our observed PC-VIPER signals. We also note that our estimates of the vibrational dephasing time (0.8—0.9 ps, SI Section 2.2), whilst within the same order of magnitude as our PC and PL-VIPER lifetimes (0.26—0.3 ps), nevertheless differ significantly by more than the sum of the measurement and fitting uncertainties. This casts further doubt on VSFG as the mechanism behind our observed PC/PL-VIPER signals.

Supplementary Note 6: Wavelength Dependent Control Measurements

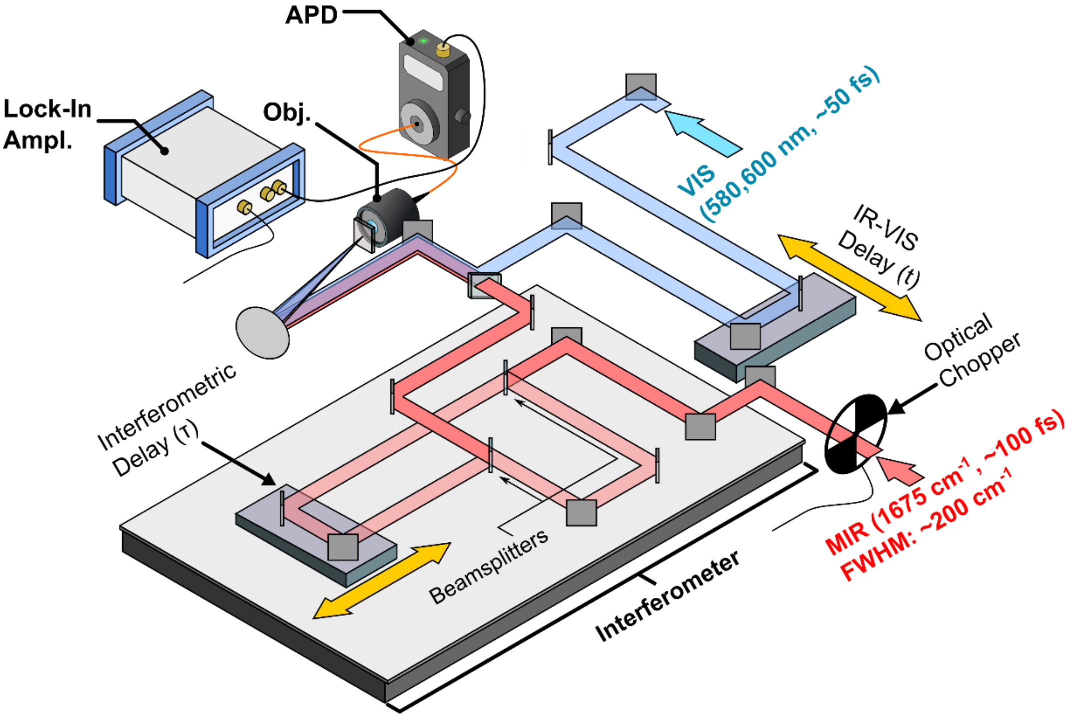

Supplementary Figure 8: Schematic of the wavelength-dependent control experiments (Obj.: Microscope Objective, APD: Avalanche Photodiode)

We perform an additional wavelength-dependent control experiment to demonstrate that the long-lived feature in our material cannot be ascribed to a sum-frequency effect. The setup for our control experiment is shown in Supplementary Figure 8. We employ a microscope objective with a working distance of 20 mm and a NA of 0.4 to detect our PL, leading to a detection half-angle of approximately 23.6 degrees. The experiment is analogous to our PL-VIPER experiment described in the main text, with one key difference: in these experiments, we detect emission along a collinear geometry with respect to the incident IR and visible pulses. In this case, we expect that not only PL to be collected by our detection system but also VSFG (and other non-linear processes that propagate along the phase matching direction), should they exist. The selection of either PL and/or VSFG can be achieved by tuning the pump wavelength. In our first experiment, we select a pump wavelength that would be expected to populate the electronically excited state (either via our VIPER double resonance effect or via the absorption of sum-frequency photons), and so produce photoluminescence, which coincides with our chosen spectral detection window. This is essentially a repeat of the PL-VIPER experiments described in the main text. In the second experiment, we choose a visible pulse wavelength that is too long to stimulate the electronic transition even in the presence of vibrational pre-excitation (either *via* the VIPER double resonance effect or via absorption of sum frequency photons), but whose sum-frequency photons would now coincide exactly with our detection window (See Fig. S13). In this case, we expect to see no photoluminescence, but would be sensitive to the generation of any sum-frequency photons.

There results of control experiments are shown in Supplementary Figure 9. The left column shows the PL-VIPER data measured in the collinear geometry, employing a visible wavelength of 570 nm. The results are analogous to Figure 3a and 4a of the main text – we observe a single vibrational mode with a sub-ps lifetime. The only difference is the presence of an additional strong and short-lived signal within pulse overlap period (likely due to the contamination of PL-VIPER channel by multiphoton responses). The left-bottom panel shows that time kinetic can be fitted well by combination of 300-fs decay and 10-fs (instant) decay, convoluted with a ~100 fs Gaussian response function.

The right column presents our attempt to directly measure a VSFG signal by shifting the pump spectrum by 30 nm to the red. In this case, the PL-VIPER signal should be strongly suppressed as the combination of sub-bandgap photon and vibrational quanta is insufficient for excitation of the electronic state. However, the VSFG signal (if any) should be observed in the detection window of the bandpass filter used. In this experimental arrangement we don't observe any evidence of VSFG signal but only evidence of multiphoton effects within the pulse overlap. No signal at all is observed beyond a delay of 300fs. These results confirm that the observed 300-fs kinetic can be attributed to PL-VIPER process, while the effect VSFG generation and absorption is negligible.

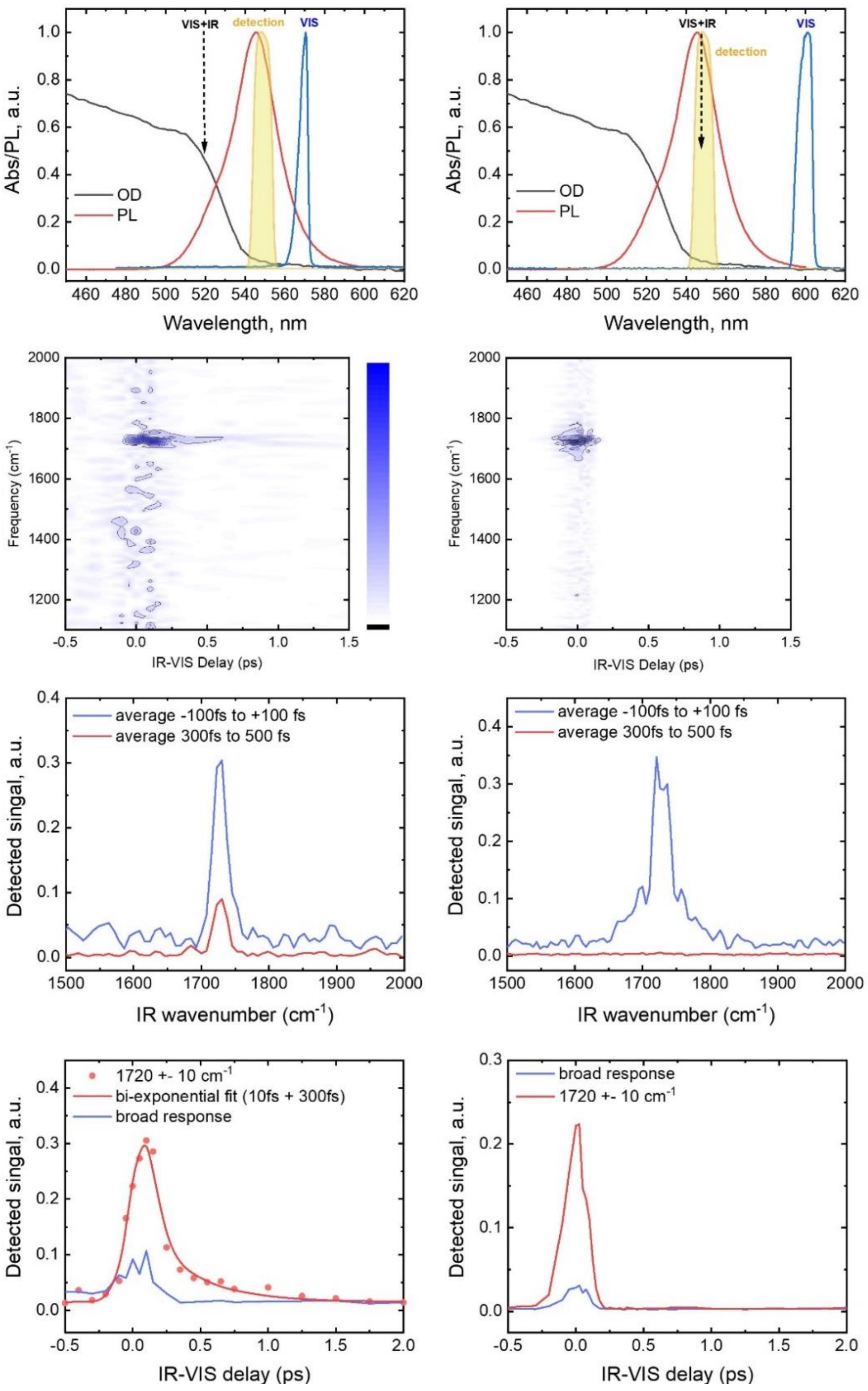

Supplementary Figure 9: Results of control experiments conducted using a 570 nm visible pulse (left column) and a 600 nm visible pulse (right column). From top to bottom: absorption and PL spectra of the $FAPbBr_3$ film employed in this experiment, including the spectrum of the visible pulse (blue), the detection window determined by our choice of bandpass filter (shaded orange), and the wavelength of any photons produced via VSFG; (top-middle) 2D heatmaps obtained under our two excitation conditions showing clearly the presence of a long-lived signal only under 'resonant' conditions; (bottom-middle) spectra obtained by integrating over the pulse overlap time (blue), and after the pulse overlap time (red); (bottom) kinetics of the 1720 cm^{-1} feature under both excitation conditions.

Given both the N-C=N stretch and the NH bending modes covered by our IR pulse are both IR and Raman active and thus could generate VSFG signals, it may be surprising that we observe no VSFG signal in our thin films, however we identify two reasons why VSFG is unlikely to be significant in $FAPbBr_3$:

1) At room temperature, $FAPbBr_3$ is known to adopt the centrosymmetric $Pm\bar{3}m$ space group; consequently, VSFG within the bulk material is impossible.

2) While VSFG at the interfaces of the $FAPbBr_3$ layer(s) can occur despite the centrosymmetricity of the bulk material, this would require an ordering of the dipoles of the interfacial formamidinium cations. This in turn requires the natural rotational freedom of the formamidinium species to be suppressed such that they are orientationally ordered. Experiments on methylammonium perovskites point towards this confluence of factors being the exception, rather than the rule in these materials.[1]

Supplementary Note 7: Ab Initio Simulations

Out of equilibrium AIMD: To compare more directly with VIPER experiments, AIMD was performed at 300 K where the antisymmetric stretching mode is vibrationally excited. An ensemble of trajectories was initialized with both atomic positions and velocities taken from the equilibrium MD trajectory described in the previous section. One FA molecule out of the eight molecules in the supercell was selected and its atomic velocities were increased along the antisymmetric stretching mode such that the total kinetic energy was increased by three quanta of the vibrational mode (600 meV). This larger excitation was used to increase the signal from the vibrational mode against the large thermal fluctuations of the lattice, but the qualitative results are relatively insensitive to the details of the excitation. AIMD was performed from these starting configurations in the microcanonical (NVE) ensemble with a time step of 1 fs, and the system was evolved for 2.5 ps.

The vibrational modes were calculated using density functional perturbation theory (DFPT) to calculate the phonon modes of the $FAPbBr_3$ unit cell. The phonon mode at 1707 cm^{-1} corresponded to an antisymmetric stretching mode largely confined to the FA cation, in good agreement with spectroscopic results. The atomic displacements associated with this mode are then translated and rotated to align with the selected FA cation when adjusting velocities of initial structures.

For each trajectory, the frequency-dependence of the band gap dynamics were calculated using Welch's method with a Hann window with a length of 400 samples and a 50 % overlap. The results from five nonequilibrium trajectories were then averaged to obtain the results in Fig. 5.

Hydrogen bond dynamics: We define a hydrogen bonding event as the point where the H•••Br bond length $R_j(t)$ significantly shortens and experiences a minimum ($dR_j/dt = 0$, $d^2R_j/dt^2 > 0$) during an interval when $R_j(t) \leq r_0$ for longer than 45 fs (Fig S7b). We chose r_0 = 2.65 Å based on the first peak in the H-Br radial distribution function (Fig S7a). To calculate the bond length evolution after a bonding event, we calculate the interatomic distance between each N-H hydrogen on the FA molecule and each of the Br atoms in the surrounding inorganic cage. For each H•••Br pair, we identify intervals satisfying the above requirements and identify the time t_0 in each interval where the bond length of the identified pair is smallest. We identified 3826 bonding events between the eight FA cations in the supercell and the inorganic cage during the 50 ps simulation. The distance between the pair of atoms was then tracked for times $t_0 + \tau$ after the bonding event and the bond length evolution was averaged over all identified bonding events (Fig. 5b and Fig. S8).

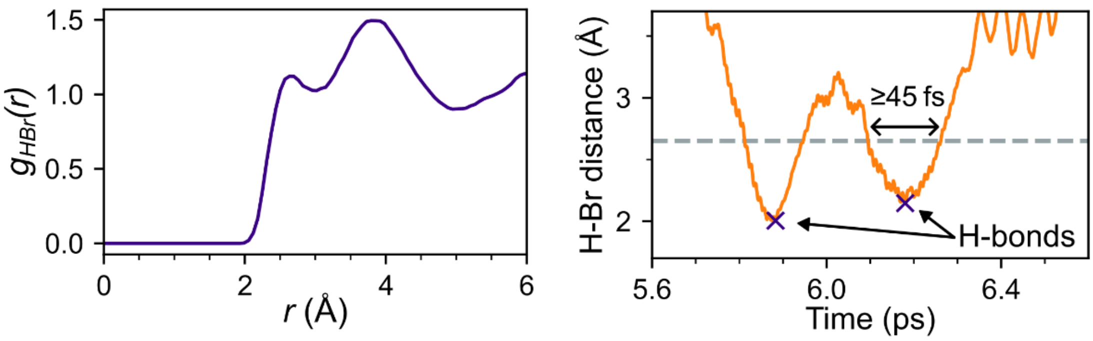

Supplementary Figure 10: (a, left) Radial pair distribution function between H and Br. (b, right) Schematic for identifying hydrogen bonding events. Dashed line is at 2.65 Å.

Dynamics from *Ab Initio* Calculations

The AIMD simulation shows highly transitory bonding dynamics. The H•••Br interactions manifest in swift decreases in the distance between one pair of atoms. The bond length begins to increase again almost immediately, and the atoms spend almost no time in a "bound" state, showing that the interactions between the FA cations and the inorganic cage are highly dynamic (Fig. S8). To quantify this behavior, we track the change in H•••Br bond length following a hydrogen bonding event as described in the previous section. This analysis shows a rapid increase in the bond length, which is fit to a biexponential with decay times of 280 fs and 2 ps (Fig. S9). This corresponds well to the two-timescale picture put forward in previous works. The shorter timescale corresponds to fast translational and rotational "wobbling" of the FA cations and is likely associated with the initial decoupling of the FA cation from the lattice observed in the VIPER experiment. The longer timescale corresponds to larger cation reorientation where the FA rotates to bind with different Br atoms, and can be seen in the large, slow changes in the H-Br distance in Fig. S8.

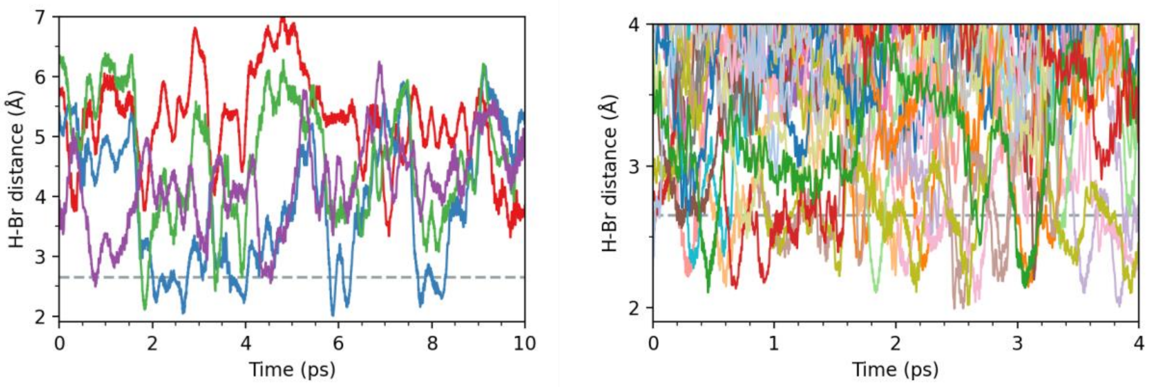

Supplementary Figure 11: H-Br distances for all possible bonds between on FA ion and one Br atom (a, left), and for all possible H-Br pairs involving one FA cation and any neighboring Br atom (b, right). The dashed line at 2.65 Å defines the cutoff for H-bonds. The interactions show transitory behavior, and no long-lived bonds form.

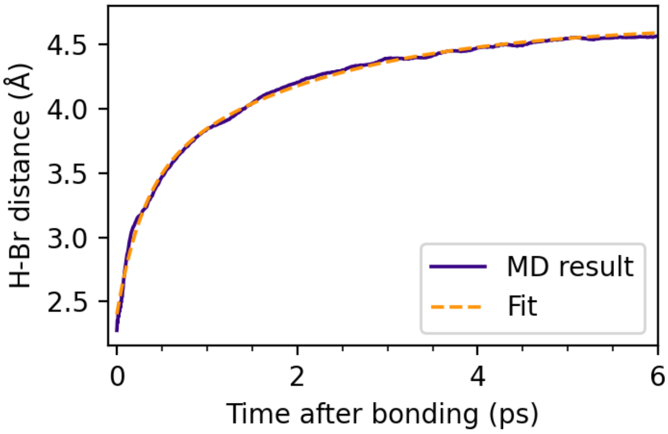

Supplementary Figure 12: Ensemble averaged evolution of the H-Br bond distance following a hydrogen bonding event.

Supplementary Note 8: Experimental origins of the coherent stimulation of the ν(NCN) mode

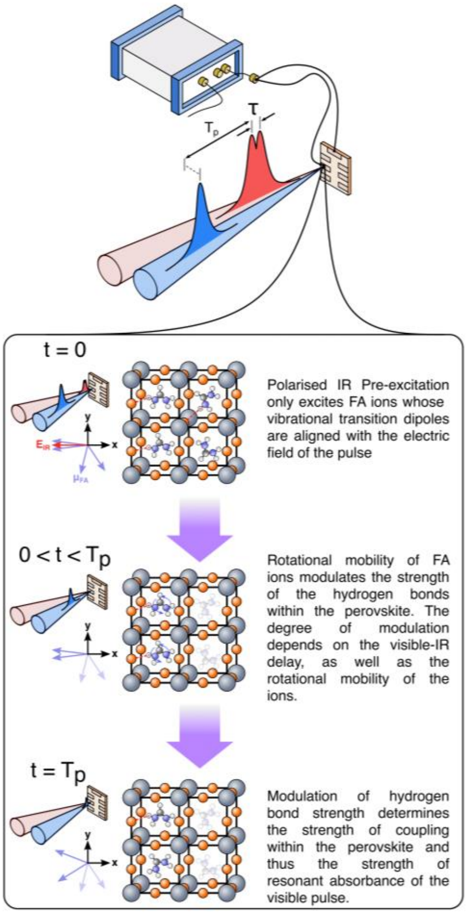

Supplementary Figure 13 Illustration of the orientational selectivity of a polarized IR pulse and the effect of cation rotation on the hydrogen bond dynamics

The use of linearly polarized IR pulses both in our PC and PL-VIPER experiments will predominantly stimulate those vibrational modes whose transition dipole moments are aligned with the electric field of our IR pulse; the absorption being proportional to $\cos^2\theta_{\vec{\mu}\vec{E}}$, where $\theta_{\vec{\mu}\vec{E}}$ is the angle formed between the transition dipole moment of the 1700 cm^{-1} mode and the electric field of the IR pulse. Thus, rather than probing an isotropic distribution of cation orientations, our IR pulse naturally selects an ensemble of molecular modes that are oriented in a common direction, as shown in Fig. S10(a). We note that polarization resolved time-resolved IR experiments have been used to probe orientational dynamics in a variety of hybrid perovskite systems[3,4]. From this initial highly organized ensemble of FA ions, we expect random rotational diffusion processes to 'scramble' their initial orientations. Given that hydrogen bonds are expected to be highly sensitive to the orientation of the FA ions, we anticipate that this scrambling of molecular orientations will modulate the molecular bandgap and so lead to the kinetics observed in our PC-VIPER and PL-VIPER experiments.

References:

[1] W. Sung et al., Preferred Orientations of Organic Cations at Lead-halide Perovskite Interfaces Revealed Using Vibrational Sum-frequency Spectroscopy, Mater. Horiz. 2020, 7, 1348-1357.

[2] K. Inoue et al., 2D Heterodyne-detected Sum Frequency Generation Study on the Ultrafast Vibrational Dynamics of H_2O and HOD Water at Charged Interfaces, J. Chem. Phys. 2015, 142, 212431.

[3] A. Bakulin et al., Real-Time Observation of Organic Cation Rotation in Methylammonium Lead Halide Perovskites, J. Phys. Chem. Lett. 2015, 6, 18, 3663–3669

[4] N. P. Gallop et al., Organic Cation Dynamics in Metal Halide Perovskites : Effects on Phonons and Material Properties, J. Phys. Chem. Lett. 2018, 9, 20, 5987–5997
